# Early Detection of Nucleation Events From Solution in LC-TEM by Machine Learning

**DOI:** 10.3389/fchem.2022.818230

**Published:** 2022-01-24

**Authors:** Hiroyasu Katsuno, Yuki Kimura, Tomoya Yamazaki, Ichigaku Takigawa

**Affiliations:** ^1^ Institute of Low Temperature Science, Hokkaido University, Sapporo, Japan; ^2^ RIKEN, Center for Advanced Intelligence Project, Tokyo, Japan; ^3^ Institute for Chemical Reaction Design and Discovery (WPI-ICReDD), Hokkaido University, Sapporo, Japan

**Keywords:** transmission electron microscopy, object detection, nucleation, machine learning, YOLOv5

## Abstract

To support the detection, recording, and analysis of nucleation events during *in situ* observations, we developed an early detection system for nucleation events observed using a liquid-cell transmission electron microscope. Detectability was achieved using the machine learning equivalent of detection by humans watching a video numerous times. The detection system was applied to the nucleation of sodium chloride crystals from a saturated acetone solution of sodium chlorate. Nanoparticles with a radius of more greater than 150 nm were detected in a viewing area of 12 *μ*m × 12 *μ*m by the detection system. The analysis of the change in the size of the growing particles as a function of time suggested that the crystal phase of the particles with a radius smaller than 400 nm differed from that of the crystals larger than 400 nm. Moreover, the use of machine learning enabled the detection of numerous nanometer sized nuclei. The nucleation rate estimated from the machine-learning-based detection was of the same order as that estimated from the detection using manual procedures.

## 1 Introduction

The nucleation of crystals is the first stage of crystallization and the origin of all materials. Because materials have a wide range of applications, including metals, chemical compounds, and biological materials, numerous studies of the crystallization of materials have been conducted over the past 50 years. The general understanding of crystallization is summarized in the classical nucleation theory (Markov, 2003). In the classical nucleation theory, only the exchange of single growth units is considered and there is only one nucleation pathway. The simple classical nucleation theory provides important basic concepts, such as a critical nucleus, and observable physical quantities, such as the nucleation rate. The validity of the nucleation theory on an atomic scale has been studied through the two-dimensional epitaxial growth of metals and semiconductors using a combination of molecular beam epitaxy and scanning probe microscopy (Michely and Krug, 2004).

Over the past decade, nonclassical nucleation phenomena in liquids have attracted attention of researchers because the proposed hypotheses are based on both experimental results (Yoreo et al., 2015; Lee et al., 2016; Ishizuka et al., 2017; Driessche et al., 2018) and computational results (Tanaka et al., 2017; Takahashi et al., 2021). Liquid cell transmission electron microscopy (LC-TEM) is a recently developed *in situ* observation method (de Jonge and Ross, 2011) that has been widely used to observe nanoscale objects in liquids. The spatial and temporal resolution of LC-TEM has been improved to atomic-scale observations (de Jonge et al., 2019), similar to ordinary transmission electron microscopy (TEM) observation. Nevertheless, observing a moving object by TEM is difficult because the image changes from moment to moment. In general, one approach to obtaining a clear image is to minimize the exposure time by increasing the electron dose. The integration of an electron beam improves the signal-to-noise ratio; however, the electron beam might damage samples (Egerton et al., 2004). In the case of LC-TEM, in particular, the solution degrades because of the radiolysis of the sample solution by the electron beam (Schneider et al., 2014). The improvement by integration is not effective when the acquired image changes in response to beam effects. Observations in which the image changes, such as *in situ* observations, require clear images with a short exposure time. Thus, *in situ* observation requires a low-dose electron observation technique. Denoising methods based on sparse coding (Anada et al., 2019) and a deep neural network (Katsuno et al., 2021; Vincent et al., 2021) have been developed in addition to ordinary methods, and these methods are expected to enable observations with a low dose of electrons in the future. However, there are a few issues specific to *in situ* observation. The same image cannot be acquired twice because the acquired image constantly changes, which introduces the difficultly in *in situ* observations of requiring the operator to decide immediately what to look for and where to look when acquiring an image. When the image acquisition fails, the same experiment must be repeated. The damage to the sample by the electron beam is cumulative. To avoid accumulating damage to the sample, we propose an early detection method that uses machine learning to detect changes in the object at an early stage while it is observed at low magnification. When more detailed information is necessary, the magnification can be increased and the observation of the object can be continued.

Recently, the use of machine learning in TEM has been integrated into the automation of tasks in biology (Falk et al., 2019; Wagner et al., 2019). This research has been accelerated by image analysis without experts, resulting in a useful software package for acquiring and analyzing static images. As far as we know, no example of machine learning for *in-situ* observations has been reported. In this case, the waiting time for the results obtained by machine learning is important, as is the accuracy. One of the useful *in situ* observation methods is early object detection. Some methods of object detection in machine learning have been proposed including region-based convolution neural networks (R-CNN) (Girshick et al., 2014), the single-shot multibox detector (SSD) algorithm (Liu et al., 2016), the “you only look once” (YOLO) algorithm (Redmon et al., 2016), and the EfficientDet model (Tan et al., 2020). Although these detection methods differ from each other, they all detect objects in a rectangular region and classify the region. Semantic segmentation enabling pixel-wise detection methods such as fully convolutional networks (FCNs) (Long et al., 2017), the Mask R-CNN (He et al., 2017) can also detect objects. These methods might be useful if the time for the analysis is sufficiently long. Our interest in the present paper is object detection at a frame rate greater than 10 frames per second during *in situ* observation. From the viewpoint of early detection of events, it is sufficient to specify the region; thus, we carry out detection using a YOLO algorithm. The YOLOv5 algorithm (Jocher et al., 2021) has recently been released and has been successfully used in the detection of tomato diseases (Wang et al., 2021), the detection of signal lights for railways (Liu et al., 2021), and the detection of smoking drivers (Shi et al., 2021). All of these applications prioritize the detection speed over a reduction of the detection rate and are intended for real-time detection. In the aforementioned applications, color information also plays an important role. TEM images are grayscale, and the information contained in the image is relatively small. It is necessary to detect only differences in contrast.

In the present study, with *in-situ* observation in mind, we develop an early detection system based on machine learning for nucleation observed by LC-TEM. The achieved detectability is equivalent to the detection determined by determined by a human operator a video of recorded TEM images numerous times. It is suggested that the detection by machine learning earlier than the human’s detection in *in-situ* observation where human see the image at the first time. Our system was used to calculate the growth rate and the nucleation rate. The detailed data related to the size of detected particles reveals that two phases of nanoparticles are present. In addition, we show the results of experiments in which a large number of nucleation events occurring can be analyzed in an instant to calculate the nucleation rate.

## 2 Methods

To develop an early detection system by a CNN model, we prepared some videos acquired by LC-TEM. Our transmission electron microscope is equipped with a field-emission gun (JEM-2100F, JEOL, Tokyo) operated at an acceleration voltage of 200 kV and a CMOS camera (Flash, EM-Z15327TCMOS; JEOL, Tokyo). The sample video captured the radiolysis-induced nucleation of sodium chloride (NaCl) from a saturated acetone solution of sodium chlorate (NaClO_3_) (Yamazaki and Kimura, 2021). As Cl^−^ ions were produced from Cl $O_{3}^{-}$ ions by radiolysis, the abundant Na^+^ ions and the produced Cl^−^ ions formed a supersaturate state for NaCl crystals.

In ordinary nucleation, by lowering the temperature, the solubility is decreased. As a result, there is a difference between the dissolved amount and the solubility, and the supersaturation to solid becomes larger. In our case, the solubility remains unchanged, but the dissolved amount increase: Cl^−^ ions are produced by radiolysis of Cl $O_{3}^{-}$ ions in the solution as suggested in Yamazaki and Kimura (2021). Since the amount of Cl^−^ ions increases, the supersaturation of NaCl crystals to acetone solution increases.

When the observation was started, the electron beam was introduced into the solution, and the solution quickly becomes supersaturated. We used two sample solutions: one with and one without molecular sieves to remove residual water from the solution. Therefore, a very small amount of water (less than 0.3 wt%) was present in the sample solution without molecular sieves because the acetone solution was a special reagent grade (FUJIFILM Wako Pure Chemical Corp., Osaka). Those sample solutions were prepared in a beaker. The solution was injected into the liquid cell of the TEM holder using a syringe. The typical magnification was 2000× or 3,000×, and the dose rate was approximately (2.0–3.1)× 10^2^ electrons nm^−2^s^−1^. The frame rate of the videos was 10 fps. When we started the observation, nanoparticles of NaCl emerged immediately because of the irradiation. The shape of the NaCl particles depended on the degree of supersaturation (i.e., the experimental setup) although the crystal structure was NaCl type cubic and the crystal shape is a cube in equilibrium. In our video, two types of particles appear. The typical shape in one case is circular. The other particles are dendritic with three- or four- fold rotational symmetry because of the crystal orientation.

To prepare the training dataset, the images were extracted from the videos and annotated using LabelImg, which is an open-source program of an annotation application. To avoid false detection, we did not annotate the particles formed around the edges. We applied the standard YOLOv5 algorithm as the machine learning model to detect NaCl particles in a liquid. The images were input to the machine learning model and the output data was the positions and size of the boxes. The parameters of the model were adjusted so that the output data was fitted the annotated data. For training and validation, we used three videos that included particles with various shapes. These three videos differ from those analyzed in Section 3.

The numbers of training and validation images was 322 and 34, respectively. Because each image shows several particles, the number of labels was 1859 for training and 188 for validation. After training using the model pretrained on the COCO dataset, we obtained the model parameter for YOLOv5s, which is the simplest model in YOLOv5. The model parameters were evaluated by two simple factors. One factor is the precision score, which is the fraction of correct detections among all detections shown in Table 1:

$$Precision=\frac{TP}{TP+FP}.$$ (1)

**TABLE 1:** Precision scores [TP/(TP+FP)] in the confusion matrix.

		Detection	
		Positive	Negative
Labels	Positive	***True positive (TP) correct detection***	False negative (FN) missing labels
	Negative	*False positive (FP) incorrect detection*	True negative (TN)

The other factor is the recall score, which is the fraction of correct detections among all labels shown in Table 2:

$$Recall=\frac{TP}{TP+FN}.$$ (2)

**TABLE 2:** Recall scores [TP/(TP+FN)] in the confusion matrix.

	Detection	
	Positive	Negative
Positive	***True positive (TP) correct detection***	False negative (FN) missing labels
Negative	*False positive (FP) incorrect detection*	True negative (TN)

The precision and recall scores of validation are 0.96 and 0.92, respectively, as summarized in Table 3.

**TABLE 3:** The number of images and labels, and detection quality for validation.

Training		Validation			
No. of images	No. of labels	No. of images	No. of labels	Precision	Recall
322	1,859	34	188	0.96	0.92

## 3 Results and Discussion

### 3.1 Detection for Simple Objects

**FIGURE 1:**
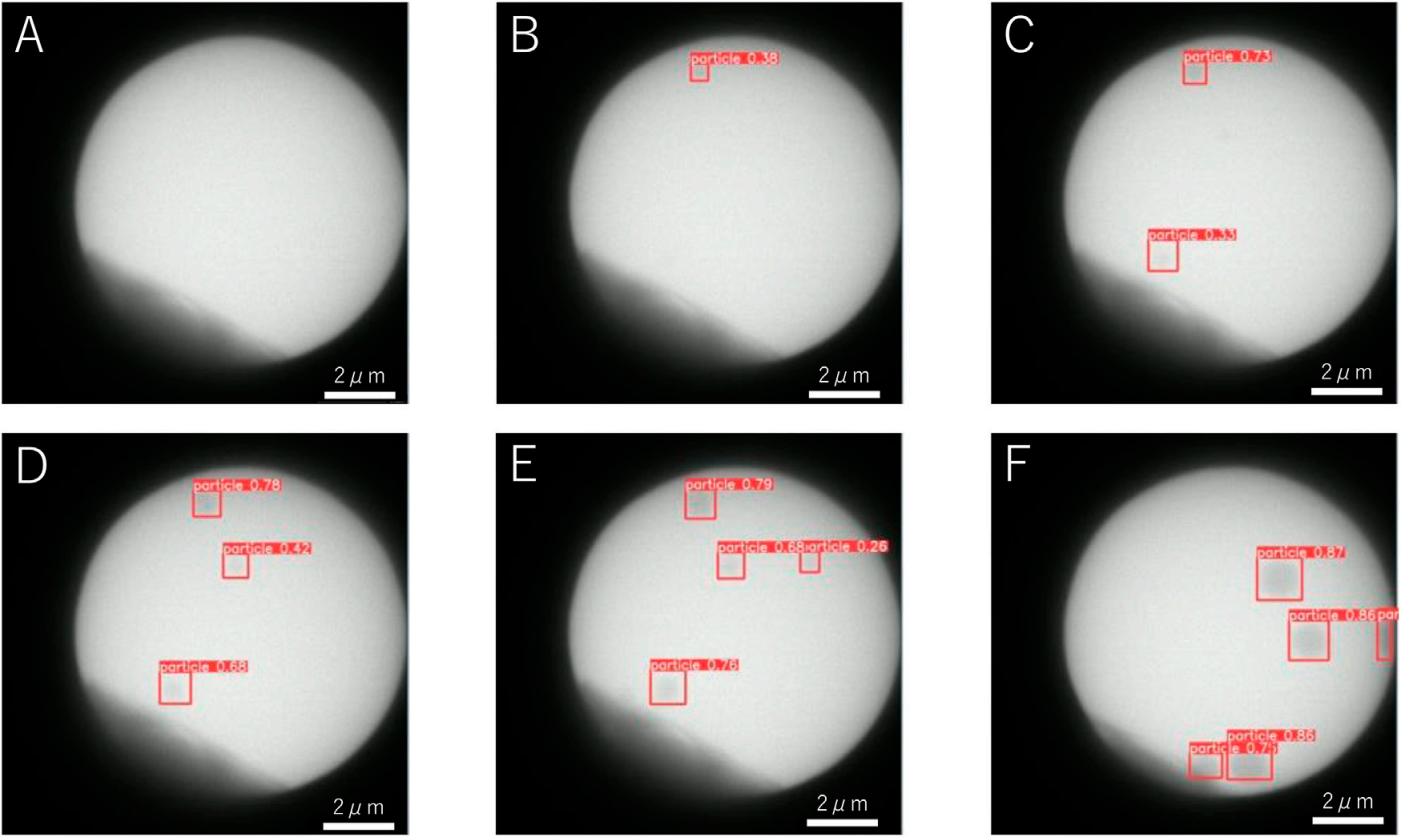
Examples of the detection of NaCl particles in a desiccant solution at **(A)** *t* = 5 s, **(B)** *t* = 19.1 s, **(C)** *t* = 22.2 s, **(D)** *t* = 24.5 s, **(E)** *t* = 28.4 s, and **(F)** *t* = 45.6 s.

We detected NaCl particles yielded from an acetone solution of dissolved NaClO_3_ powder crystals. The solution contained molecular sieves as a desiccant to eliminate residual water, which substantially lowered the amount of dissolved NaClO_3_ because of the large difference in solubility [36 g in 100 g water vs. 4.2 × 10^–5^ g in 100 g acetone (Burgess, 1978)], thereby increasing the waiting time for nucleation of NaCl crystals. The magnification was 2000×, and the dose rate was 310 electrons nm^−2^s^−1^. Examples of the detection of NaCl particles are shown in Figure 1, where the image size is 12 *μ*m × 12 *μ*m. We conducted a continuous observation, and the view was fixed at time *t* = 0 s. In the dark area at the edge of the image, the electron beam was blocked by the aperture. The bright circular region was filled with the saturated acetone solution of NaClO_3_. Until 19 s, no apparent change was observed and there was no detection (Figure 1A). The first particle was detected at *t* = 19.1 s (Figure 1B) and exhibited a radius of ∼150 nm. The second, third, and fourth particles were detected at *t* = 22.4 s (Figure 1C), *t* = 24.5 s (Figure 1D), and *t* = 28.4 s (Figure 1E), respectively. In all cases, the radius of the particles was ∼200 nm at the beginning of the detection in the 12 *μ*m × 12 *μ*m image.

**FIGURE 2:**
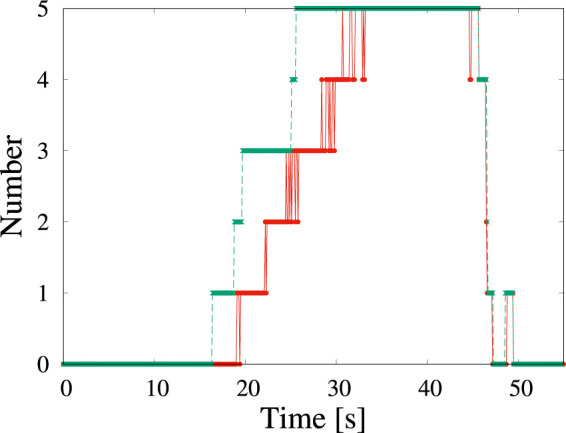
Time change of the number of particles corresponding to Figure 1 detected by machine learning (red solid line) and by the human eye (green broken line). The origin of time corresponds to when the view was fixed.

Figure 2 shows the change in the number of detected particles as a function of time. The red solid line and the green broken line show the number of detections and that of labeling by the human eye. Because the human-eye observations involve repeated observations and adjustments to the contrast, tiny particles can be labeled. The machine learning algorithm used in present study can detect particles with a delay of ∼3 s compared with the data labeling by the human eye. The delay in detection by the standard YOLOv5 algorithm is comparable to the delay when a person observes a particle when watching a video directly the first time.

In the video, the view is almost motionless until *t* = 45 s. At *t* > 45 s, the view shifts to the upper left. Thus, the particles appear to have moved to the lower right, as shown in Figure 1F. The number of particles in the image also decreases. Even though part of the particle is hidden by the aperture and its shape appears to not be circular, it is still detected. Immediately before *t* = 50 s, one half-moon-shaped particle is detected momentarily in the video.

Although some detection failures occurred, no false positive were detected. In this video, the total recall score was 0.82 (Table 4). When particles became large (40 s <t<50 s), the recall score was 0.93. This recall value is approximately the same as that of the validation data shown in Table 3.

**TABLE 4:** Numbers of images and labels and the detection quality.

No. of images	No. of labels	No. of detections	Precision	Recall
288	1,282	1,053	1	0.82

**FIGURE 3:**
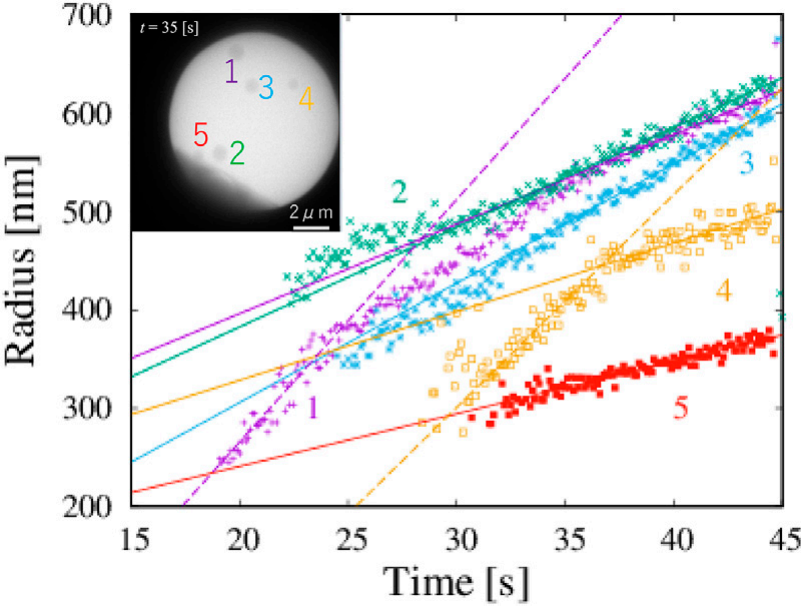
Five NaCl particles and change in radius with time, which corresponds to Figure 1. Solid lines are obtained by fitting in the range 35 s <t< 45 s for each particle. Broken lines are obtained by fitting radii less than 400 nm for particles 1 and 4. The inset shows a TEM image acquired after all five particles had formed (*t* = 35 s). Numbers represents the order of appearance.

From the obtained data of corresponding to the detected boxes, we obtained time changes of the radii of five particles (Figure 3). Each box is slightly larger than their corresponding particles. The systematic deviation does not strongly affect the study of its time change. The shape of particles is assumed to circular, and the radius of particles is estimated on the basis of the geometric average of one-half the height and one-half the width of the box. The growth rates of each particle were obtained by fitting the data in the region 35 s <t< 45 s (solid lines in Figure 3); these values are summarized in Table 5. The radius of the fifth particle may not be accurate because the window prevented us from observing the entire particle. The average growth rate excluding the fifth particle was 9.5 nm s^−1^.

**TABLE 5:** Growth rate of each nanoparticle.

Particle	Growth rate (nm s^−1^)	Growth rate in small size (nm s^−1^)
1	9.1	24.7
2	10.1	—
3	12.1	—
4	7.0	21.6
5	5.3	—
Average (1–4)	9.5	—
Average (1, 4)	—	23.1

The growth rate *V*(*r*) can be predicted by classical nucleation theory. When a circular particle with radius *r* grows in a system, the growth rate *V*(*r*) is obtained as

$$V\left(r\right)=V_{\infty }\left(1-\frac{r_{\text{c}}}{r}\right),$$ (3)

where *r*_c_ is the critical radius and *V*_*∞*_ is a constant, that depends on the concentration and the temperature. When the radius of the particle is larger than the critical radius, the growth rate is close to a constant asymptotically. The radius increases linearly with time. From our data, the value of the growth rate *V*_*∞*_ is ∼9.5 nm s^−1^.

The growth rate *V* of the stem of the NaCl crystals under the dose rate *F* is written as *V* = *K*′*F* ^1/2^, where the effective kinetic coefficient *K*′ is ∼8.5 electron^−1/2^ nm^2^ s^1/2^ (Figure 6 in Yamazaki and Kimura (2021)]. When the dose rate is substituted as 310 electrons nm^−2 s−1^, the growth rate is obtained as 150 nm s^−1^, which is very different from our observed growth rate of 9.5 nm s^−1^. The effective dose rate appears to be reduced by the presence of molecular sieves in the solution. Because the crystal shown in Figure 6 in Yamazaki and Kimura (2021) is sufficiently large, the effective dose rate in Figure 1 is estimated as 1.2 electrons nm^−2^s^−1^, using a obtained growth rate of 9.5 nm s^−1^. Thus, the effective electron dose rate becomes 1/250 as a result of molecular sieves.

In classical nucleation theory, small particles grow slower than large particles because of the surface tension, which is well known as the Gibbs-Thomson effect. However, our data for particles 1 and 4 suggest the opposite trends: the growth rate of a small particle is larger than that of a large particle. The growth rates of particles 1 and 4 with radii less than 400 nm are 24.7 nm s^−1^ and 21.6 nm s^−1^, respectively. The magnitude of the growth rate of particles with a radius smaller than 400 nm is twice larger than that of the same particle with a radius larger than 400 nm. The particles do not rotate because they are formed on a membrane. In addition, our observed particle is approximately circular, and the orientational dependence of the growth rate is negligible. The growth rate is almost the same for particles 1 and 4, even though the timing of their formation is different. The degree of supersaturation does not change through the volume diffusion of materials.

There are two possibilities of the change of the growth rate. One is the occurrence of the phase transition of a particle at the radius of 400 nm. Another is the heterogeneous nucleation of the stable phase on the metastable phase. Our observations do not allow us to distinguish between them. We speculate that the non-linear function arises from the crossover between the growth of the metastable phase and the growth by the heterogeneous nucleation of the stable phase on the surface of the metastable phase. Thus, the larger growth rate indicates that a metastable phase appears in the early stages of the nucleation process. This conclusion suggests that the nucleation of NaCl from a saturated solution of NaClO_3_ in acetone is not a single-phase process. We therefore found an example of the two-step nucleation process of simple circular precipitates.

### 3.2 Detection for Objects With Complex Shape

We investigate the detectability of dendritic crystals that exhibit rapid growth. The magnification was 3,000× and the dose rate was 2.0 × 10^2^ electron nm^−2^s^−1^. The solution was the same as that studied in the previous section but without the addition of molecular sieves. The outline of the video is as follows: The observation starts at *t* = 0 s and many nanoparticles emerge within 1 s. At *t* = 1 s, the nucleation of nanoparticles is almost completed and each nanoparticle grows. Moreover, nanoparticles also coalesce with their neighbors. The production of nanoparticles stops because the solute is consumed by the nucleation and growth of the nanoparticles.

**FIGURE 4:**
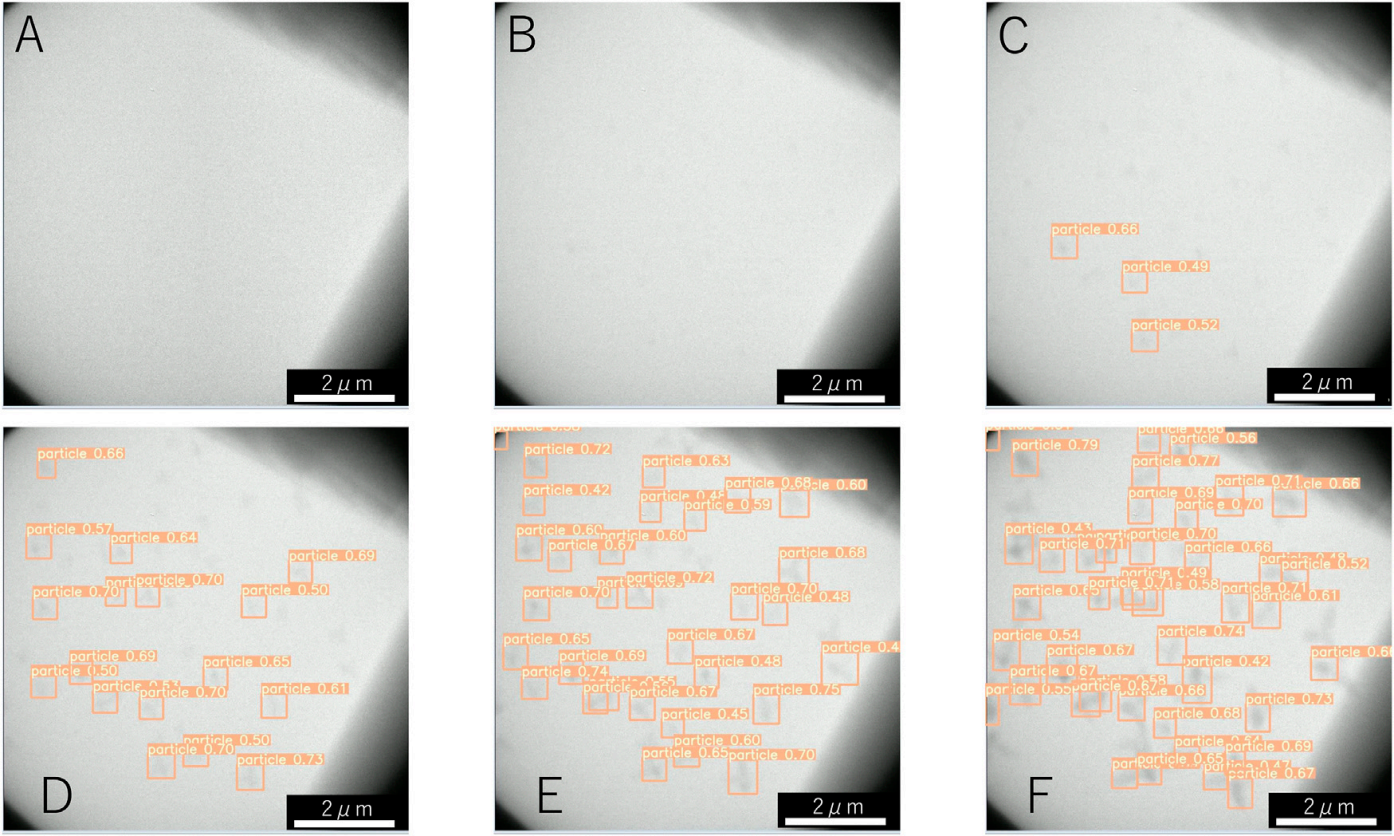
Detection of NaCl particles with dendritic shape by machine learning at **(A)** 0.1 s, **(B)** 0.4 s, **(C)** 0.6 s, **(D)** 1.5 s, **(E)** 2.5 s, and **(F)** 5.5 s. The time of 0 s corresponds to the start of electron irradiation.

**FIGURE 5:**
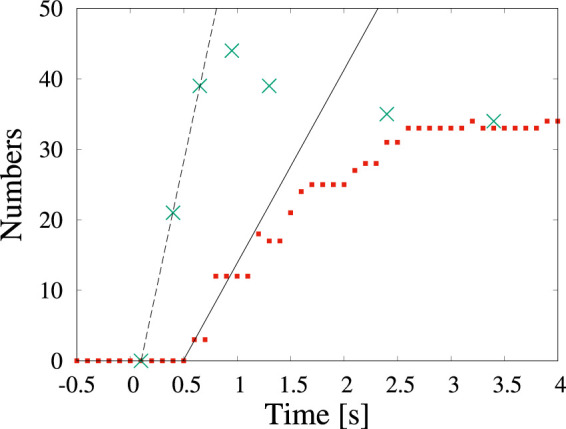
Time change in the number of particles counted by detection (red squares) and by the human eye (green cross) in Figure 4. The time of 0 s corresponds to the start of observation.

Examples of the detection by machine learning of dendritic crystals are shown in Figure 4. Here, *t* = 0 s corresponds to when the shutter was opened. Immediately after the shutter was opened (Figure 4A), no objects were observed. At *t* = 0.4 s, numerous tiny particles emerge although there is no detection by machine learning (Figure 4B). Our detection by machine learning often overlooks small nuclei. The first detection is at *t* = 0.6 s (Figure 4C). As time passes, the number of detections increases (Figures 4D–F). To check the detectability, we counted the number of dendritic crystals by adjusting the contrast. Figure 5 shows the numbers of particles suggested by YOLOv5 (red square) and counted by the human eye (green cross). The numbers increase in the period *t* < 1 s, and a difference is observed between the result obtained by YOLOv5 and that by the human eye. Both results show that the number of particles was steady at *t* > 3 s, and both results are consistent. In the steady region (*t* > 3 s), the dendritic crystals become large and are clearly observed and the change in the image is relatively small. In this case, the number of dendritic crystals counted by YOLOv5 is consistent with that of counted by the human eye. During steady growth, the standard YOLOv5 algorithm can detect crystals at the same level as the human eye, as mentioned in the preceding subsection.

As we characterize the change of the number of particles over time, we can estimate the nucleation rate. The nucleation rate indicates how many nuclei are yielded per unit area and unit time. The observed nuclei appear on amorphous silicon nitride membranes on the top and the bottom of a silicon tip (Yamazaki and Kimura, 2021). The nucleation occurs in two dimensions and the area is ∼ 88 *μ*m^2^, when both the top and bottom surfaces of the membrane are considered. By fitting the data, we can estimate the nucleation rate as 0.3 *μ*m^−2^s^−1^ (solid line) and 0.8 *μ*m^−2^s^−1^ (broken line) on the basis of detection using the standard YOLOv5 algorithm and detection by the human eye, respectively. Although the number of particles by YOLOv5 is less than one-half of that detected by the human eye, the estimated nucleation rates are of the same order. In most discussions concerning crystallization, determining the order of the nucleation rate is sufficient. If we need to estimate physical quantities such as the nucleation rate more accurately, improving the detectability by preprocessing the images is worthwhile.

In the preceding subsection, we detected circular shaped particles in a video in which the particles slowly changed. In this subsection, we detect dendritic particles whose size and shape change rapidly under a dose rate of 2.0 × 10^2^ electron nm^−2^s^−1^. As reported in (Yamazaki and Kimura, 2021), the growth rate depends on the dose rate in a solution without molecular sieves. Although the dose rate is smaller than that in Figure 1, numerous dendrites are formed. The shape of the nanoparticles depends on the degree of supersaturation. When the degree of supersaturation is low, the shape is similar to the equilibrium shape, which is compact. When the degree of supersaturation is high, the shape becomes dendritic because fast growth proceeds before sufficient relaxation of the shape can occur. Because the amount of water is related to the precipitation of NaCl crystals, the effect of water loss due to molecular sieves strongly affects the degree of supersaturation. The decrease in the degree of supersaturation is attributed to a decrease in the amount of dissolved chlorate ions, which become chloride ions (Yamazaki and Kimura, 2021). In Figure 2, we observe the appearance of five particles in the 126 *μ*m^2^ solution area in 10 s and the nucleation rate is estimated as 0.004 *μ*m^−2^ s^−1^. This rate is 200 times smaller than the value obtained in Figure 4. It is a conventional result because of the same aforementioned conclusion deduced from the crystal shape.

When a large number of nuclei are produced in a small area, the growth rate becomes small because the growth of each nucleus competes with other nuclei on the supplied material. Although this effect should be studied in detail according to the rate-limiting process, the limiting process of the present system, including the radical reaction, is unclear. However, the influence of the surrounding nuclei is relatively weak immediately after the nucleation occurs. From the classical nucleation theory, the nucleation rate *J* is written as *J* = ω*Γ*Z*_1_  exp (−ΔG*/*k*_B_ *T*) where *ω* is the frequency of the attachment of atoms to the critical nucleus, Γ is the Zeldovich factor, *Z*_1_ is the steady-state concentration of a monomer, and ΔG* is the free energy of the critical nucleus (Markov, 2003). The pre-exponential factor *A* = ω*Γ*Z*_1_ is not sensitive to the supersaturation in comparison with   exp (−ΔG*/*k*_B_ *T*) = exp (−*B*/Δ*μ* ^2^). Therefore, we assume

$$J=A\exp\left(-\frac{B}{\Delta \mu^{2}}\right),$$ (4)

where *B* = 16*πσ* ^3^ *v*/(3*k*_B_ *T*), *σ* is the interfacial energy, *k*_B_ is the Boltzmann constant, and *T* is the temperature. The ratio of the nucleation rate in Figure 1 (*J*_1_) to that in Figure 4 (*J*_4_) is

$$\frac{J_{1}}{J_{4}}=\exp\left[-\left(\frac{B_{1}}{\Delta \mu_{1}^{2}}-\frac{B_{4}}{\Delta \mu_{4}^{2}}\right)\right]≃\exp\left(-\frac{B_{1}}{\Delta \mu_{1}^{2}}\right),$$ (5)

where Δ*μ*_1_ and Δ*μ*_4_ are the chemical potential of the solid phase in Figures 1, 4, respectively. Here, we ignore the term Δ*μ*_4_ because the effective dose rate in Figure 1, as discussed in the previous subsection is sufficiently smaller than that in Figure 4. The lower limit of the ratio of the interfacial energy and the chemical potential is obtained as

$$\frac{B_{1}}{\Delta \mu_{1}^{2}}≃\ln\left(\frac{J_{4}}{J_{1}}\right)≃5.3,$$ (6)

where *B*_1_ depends on the interfacial energy of an unknown phase discussed in the previous subsection and *J*_1_ is the effective dose rate (1.2 electrons nm^−2^ s^−1^).

In Figure 2A of Yamazaki and Kimura (2021), two NaCl crystals appears in an area of 22 *μ*m^2^ in 10 s with an electron dose rate *J*_2_ = 37 electron nm^−2^ s^−1^. The crystal shape is dendritic, and its symmetry is reflected in the crystal symmetry. The precipitate is assumed to be crystalline, and it is not identical to that in Figure 1 because the particles are circular. Using the same approach, we obtain the relation of the interfacial energy *B*_2_ and the chemical potential to the solid Δ*μ*_2_ as

$$\frac{B_{2}}{\Delta \mu_{2}^{2}}≃\ln\left(\frac{J_{4}}{J_{2}}\right)≃4.5.$$ (7)

*B*_2_ depends on the interfacial energy of the solid phase of the NaCl crystal. Because the chemical potential Δ*μ* depends on the 1/2 power of the dose rate, we can estimate the difference of the interfacial energy of the unknown phase *σ*_1_ and crystal phase *σ*_2_ as *σ*_1_ ≃ 0.33*σ*_2_. In general, the interfacial energy of a metastable phase is smaller than that of a stable phase; therefore, metastable crystals nucleate before stable crystals. The present system also conforms to this empirical rule.

We attempted to identify the unknown metastable phase of NaCl shown in Figure 1. The possible structures for the NaCl crystals are the NaCl-type structure and CsCl-type structure, and the NaCl-type is observed in the stable phase. The ratio of the interfacial energy of the CsCl-type to that of the NaCl-type can be roughly estimated by a bond counting method. On the (100) surface of the NaCl-type structure, the number of Na^+^ ions and Cl^−^ ions is 2 and 2, respectively, in a unit cell with a lattice constant 5.6 Å. On the (100) surface of the CsCl-type, the number of Na^+^ ions and Cl^−^ ions are 0.5 and 0.5, respectively, in one unit cell. Its lattice constant is assumed to be 3.3 Å on the basis of the ratio of the lattice constant of NaCl-type to CsCl-type of other crystals such as CsCl crystals and CsBr crystals (Kimura et al., 2002). The ratio of the interfacial energy of CsCl-type to NaCl-type is ∼0.71. Because the ratio of the interfacial energy of the unknown phase shown in Figure 1 to that of the NaCl crystal shown in Figure 4 is 0.33, the interfacial energy of the unknown phase is inconsistent with that of a CsCl-type NaCl crystal. In conclusion, we inferred that the unknown phase observed in Figure 1 is an amorphous phase of NaCl or dense-liquid after liquid-liquid phase separation because of the large growth rate, the circular shape, and the small interfacial energy.

Object detection algorithm have difficulty in detecting small objects, and first detection is inevitably delayed. Here, the difference is only 0.2 s, which is shorter than the detection delay in the previous section, where particles nucleated slowly. However, object detection is adequate for early detection because some recent TEM cameras can record several seconds before using the lookback function. By integrating the detection system into a transmission electron microscope, rare events such as nucleation phenomena will be captured within the limited resources for recording. The time necessary for the detection is within 10 ms without the CPU-GPU data transfer. Because the time required for real-time detection, which depends on the PC configuration, is approximately 15–35 ms including the CPU-GPU data transfer (Katsuno et al., 2021), object detection of nucleation phenomenon in *in situ* observation is possible when the image data are received directly through the software of a TEM camera.

## 4 Summary

We proposed a method for the early detection of nucleation phenomena using TEM observations in conjunction with the machine learning. For the detection method to be used in *in situ* observations, the standard YOLOv5 algorithm was adopted.

The detection method was applied to the nucleation of NaCl crystals from a solution of acetone and NaClO_3_. Although the detection of emerging particles sometimes failed, the particles with isolated circular shapes were detected even if particles moved.

We also obtained detailed information on local stochastic process such as the time change of the size of individual particles.

The growth rates of each particle were calculated from the detection data. The results suggested that nanoparticles with a radius smaller than 400 nm and those with a radius larger than 400 nm were different phases. Nanoparticles with a dendritic shape were also detected. Although the detection rate was slightly lower, the estimated nucleation rate was of the same order as that estimated using manual procedures, where the images are preprocessed and then nanoparticles are counted. The preprocessing of the images enables quantitative evaluation with disadvantages in terms of early detection. Using the classical nucleation theory, we investigated the effect of an additive (molecular sieves). The additive lowered the degree of supersaturation. From the classical nucleation theory, the interfacial energy of the unknown phase of NaCl was estimated to be one-third of the interfacial energy of a NaCl crystal. This value is smaller than the roughly estimated interfacial energy of a NaCl crystal with a CsCl-type structure. We inferred from the high growth rate, the circular shape, small interfacial energy that the observed unknown phase was an amorphous or dense-liquid phase of NaCl.

The delay in the first detection of nanoparticles was ∼ 3 s and ∼ 0.2 s for particles with a large compact circular shape and particles with a small dendritic shape, respectively. The delay could be recovered by the TEM function of a recent camera, which enabled us to record several seconds before. Because the time for detection was within 20 ms, nucleation phenomena could be observed during *via in situ* observations when the detection system is integrated into the software of a TEM camera.

The problem of the time necessary for detection will be resolved with the development of computers. We hope that the opportunity to discover new science will be found by machine learning techniques just as our finding of the two-step nucleation process.

## Data Availability

The raw data supporting the conclusion of this article will be made available by the authors, without undue reservation.
